# How Different Methodologies of Harvesting and Analysing the Samples Affect the Test Results in Determining Joint Mediators

**DOI:** 10.1155/2013/631959

**Published:** 2013-02-20

**Authors:** Ibrahim Yilmaz, Nevzat Selim Gokay, Rifat Bircan, Gamze V. Saracoglu, Sergulen Dervisoglu, Alper Gokce

**Affiliations:** ^1^Tekirdag State Hospital, Turkish Republic Ministry of Health, 59100 Tekirdag, Turkey; ^2^Department of Orthopaedics and Traumatology, Namik Kemal University School of Medicine, 59100 Tekirdag, Turkey; ^3^Department of Biology, Namik Kemal University School of Science, 59100 Tekirdag, Turkey; ^4^Department of Public Health, Namik Kemal University School of Medicine, 59100 Tekirdag, Turkey; ^5^Department of Pathology, Istanbul University Cerrahpasa School of Medicine, 34099 Istanbul, Turkey

## Abstract

*Purpose*. This study has researched the affect of different methodologies of harvesting and analysing the samples in determining the mediators emerging after the rat articular cartilage injury. *Materials and Methods*. One hundred and forty-four male wistar rats were divided into 2 groups. Synovial fluid samples were taken from all of the rats. We entered into the right knees of the rats in group I (*n* = 36) under anaesthesia and took cartilage tissue samples from their distal femur. Samples were taken as reference values for enzyme linked immunosorbent assay (ELISA) and histopathological evaluations. We entered into the right knees of the rats in group II (*n* = 108) and formed complete layer of cartilage injury in their medial femoral condyles. At the end of the 15th day, the rats were sacrificed after taking synovial fluid samples from their right knees creating defect in the rats in group II. The molecular markers in the synovial fluid and cartilage tissue samples which were taken from the experimental and control groups (MMP-9, MMP-13, TIMP-1, TNF-**α**, and NO) were analysed by direct or indirect methodologies. SPSS 18.0 Package program was used in the statistical evaluation. Students *t-*test where the measurement variables between the experimental and control groups were compared was applied. Receiver Operating Characteristics (ROC) curves were used in the determination of the diagnostic sufficiency from the tissue. *Results*. No difference was found between TIMP-1 (*P* = 0.67) and MMP-9 (*P* = 0.28) levels in synovial fluid and cartilage tissue. From the molecular markers, when MMP-9, MMP-13, NO, TIMP-1, TNF-**α**′, the area under ROC curve, and *P* values were examined, MMP-13 (*P* < 0.0001, 95% CI: 0.70–0.85), NO (*P* < 0.0001, 95% CI: 0.72–0.86), and TNF-**α** (*P* < 0.0001, 95% CI: 0.91–0.98) results were found to be statistically significant. *Inferences*. The indirect ELISA protocol which we apply for the cartilage tissue as an alternative to synovial lavage fluid is a reliable method which can be used in the determination of articular cartilage injury markers.

## 1. Introduction

The molecular markers which emerge in the process after the articular cartilage injury are used in the monitoring of degenerative diseases, prognosis determination, monitoring of response to the treatment, and identification of the disease mechanism in molecular level [1, 2]. One important purpose of the measurement of molecular determinants is to examine the disease quantitatively in the early stages when the cartilage injury has not been radiologically determined yet.

Chondrocytes have a directing role in the metabolism including the construction and destruction of matrix molecules in the lifetime [3, 4]. There are simultaneous changes in tissues in the articular cartilage injury, and there is a need for molecular markers related to each tissue for the comprehensive evaluation of these changes. However, there are no official study protocols for different tissues [5]. As there can be significant differences related to biological tissues, the way of sampling of the markers which are obtained from different tissues can influence the result should be predictable. Pharmaceutical researchers have published articles on the validity of the analytical method in the determination of the method applied [5–10].

Molecular markers of the cartilage are molecules which can be in the structure of protein constituting collagen, proteoglycan, and extra cellular matrix (ECM) and they show increase and decrease in several cartilage pathologies and in the different stages of the same pathology [11]. 

Depending on the severity of the ongoing inflammation, cartilage-based markers in several intensities go out of the cell and enter into the synovial fluid. Parts of the cartilage ECM macromolecules which are poured into the synovial fluid can pass to the systemic circulation [12].

This study aims to determine the biological markers, occurring in the articular cartilage injury, directly by cartilage tissue biopsies and in synovial fluid, and present the relationship between them.

## 2. Materials and Methods

This study was conducted after the decision of the meeting dated June 1, 2010, numbered 2010/04 and the permission of the Local Ethical Committee of Experimental Animals and the Use of Live Mammal of Namik Kemal University. Experimental analyses were repeated minimum 3 times. In order to minimize the differences in the technique, anaesthesia, injury formation, and analyses were performed by the same researchers.

### 2.1. Materials

Male wistar rats were supplied from Istanbul University Experimental Medicine Research Institute. Ketamine HCl (Ketalar 500 mg) injectable 1 flacon Pfizer and xylazine (Rompun 2%) injectable solution, Parke-Davis, were used. Protein extraction solution (78510) and protease inhibitor cocktail (87785) Thermo Scientific Pierce Biotechnology, Rockford, IL & 1105, were supplied from the United States of America. NO kit was supplied from ELISA commercial kits, IL-TIMP-1 (BMS2018/BMS2018TEN) was supplied from Cayman Chemical, matrix metalloproteinase-9 (MMP-9) (BMS2016/2CE) and MMP-13 (BMS2022/BMS2022TEN) platinum were supplied from ELISA eBioscience, and TNF-*α* (KRC3011) kit was supplied from Invitrogen. Mechanical disruption was provided with EpiSonic Multi-Functional Bioprocessor 1100. Mindray MR 96 A, Chinese brand device, was used as Enzyme Lınked-Immuno-Sorbent Assay (ELISA) microplate reader. The tissues which are obtained from 144 wistar rats were studied double-blind by the same researchers.

### 2.2. Methods

One hundred forty-four male wistar rats with an average weight of 300 grams were divided into 2 groups. Synovial fluid samples were taken from all of the rats. While taking synovial fluid, the knee joint was entered by the injector and the liquid which is given into the joint was withdrawn 45 seconds later and taken into the tubes containing EDTA. Synovial fluid aspirates with PBS in the rate of 75 *μ*L 1 : 3 were centrifuged at 24°C in 3000 Revolutions Per Minute (rpm) for 15 minutes and cleared of cells and maintained at minus 40°C to be evaluated with ELISA. All of the samples were taken at 05.00 in the morning. 

The rats in group I (control group, *n* = 36) were applied arthrotomy under ketamine HCI and xylazine anaesthesia. Cartilage tissue samples were taken from distal femur medial condyles. These samples were taken as reference values for enzyme linked immunosorbent assay (ELISA) and histopathological evaluations. And then, these rats were sacrificed.

**Figure 1:**
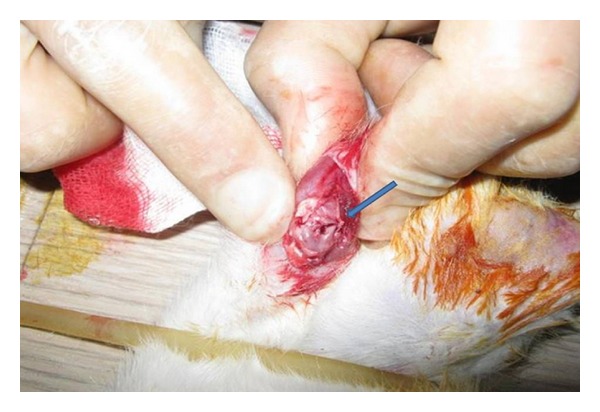
Macro view of the actualization of the cartilage complete layer injury.

The rats in group II (*n* = 108) were applied arthrotomy under ketamine HCl and xylazine anaesthesia, and a complete layer of cartilage injury was formed in their medial femoral condyles (Figure 1) [13, 14]. From the right knees of the rats where injury was formed, synovial fluid samples were taken by the same technique on the 15th day. Then, arthrotomy was applied and cartilage tissue samples were taken from their distal medial condyles. These rats were sacrificed after this operation. 

The synovial lavage fluid and cartilage tissue samples taken from the experimental and control groups were analysed by direct or indirect methodologies by MMP-9, MMP-13, NO, TIMP-1, TNF-*α*, and commercial ELISA kits which are cartilage injury molecular markers in line with company bulletins.

### 2.3. Biochemical Analyses

The samples were blindly studied. The synovial fluid tissue samples which were taken from the experimental and control groups were analysed by MMP-9, MMP-13, NO, TIMP-1, and TNF-*α* values ELISA kits and in line with the company bulletins.

**Figure 2:**
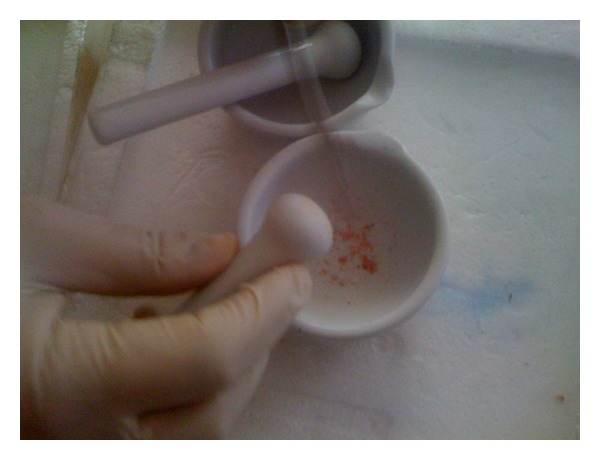
Mechanical disruption of the tissue which is frozen in the presence of liquid nitrogen.

Before conducting analyses in the cartilage tissue, the tissues were made compatible for kit study procedure. Porcelain mortar and pestle were subjected to 8% sodium hypochlorite solution. They were dried after having been washed with bidistillated water. They were wrapped with aluminium folios and kept in the drying oven at 134°C for 1 hour. At the end of this time, they were kept at −20°C for 30 minutes. The cartilage tissue samples which were broken up with and pulverized by a pestle in the presence of liquid nitrogen were taken into cryo tubes and coded (Figure 2). They were weighed at the rate of 1 : 20 (w/v) and transferred into eppendorf tubes by means of  15 no scalpel end. Primarily 400 *μ*L lysisbuffer was added on them. Then, a protease cocktail inhibitor ten times the ready volume (making 1x the last concentration) is added and vortexed for 1 minute, and centrifuging process was performed at +4°C for 5 minutes in 10.000 (g) [15, 16]. The samples were kept at −80°C for one night. For the mechanical disruption of the cartilage tissue; they were subjected to 2 times vibration and ultrasonic sound waves operation for 10 minutes under the same pressure at intervals of 20 minutes in sonicator by watering down with phosphate buffer saline (PBS; pH = 7,4) in 1.5 mL microtubes (max 400 *μ*L of sample content). And then, in line with the company bulletins, the samples were placed in the wells. MMP-9, MMP-13, TIMP-1 and TNF-*α* 450 nm, NO was evaluated by reading at ELISA microplate reader at 540 nm wave length as a result of the calculation of the data which was obtained over the nitrate and nitrite values. Standards were studies as pairs. 

### 2.4. Histopathological Analyses

In order to prove the cartilage complete layer injury, the cartilage tissue samples which were taken from each of 6 rats from both groups were also evaluated histopathologically. The samples were buried in paraffin-embedded blocks after the routine tissue monitoring. Five-micron sections were put to hematoxylin-eosin staining and examined in the light microscope (×100). Histological sections were examined in terms of 8 parameters and scored and evaluated [17].

### 2.5. Statistical Analysis

The data was evaluated by using SPSS 18.0 Package program. Descriptive statistics were calculated (mean, standard deviation) after performing data control. Students *t*-test, which is a test in the comparison of the quantitative data in the comparative analyses between the experimental and control groups, in which the measurement variables between two groups are compared, was applied. Partial correlation analysis was performed in order to explain the relationship between these mediators. Receiver Operating Characteristics (ROC) curves were used in the determination of diagnostic sufficiency from the tissue. 

## 3. Results

### 3.1. Histopathological Evaluation

**Figure 3:**
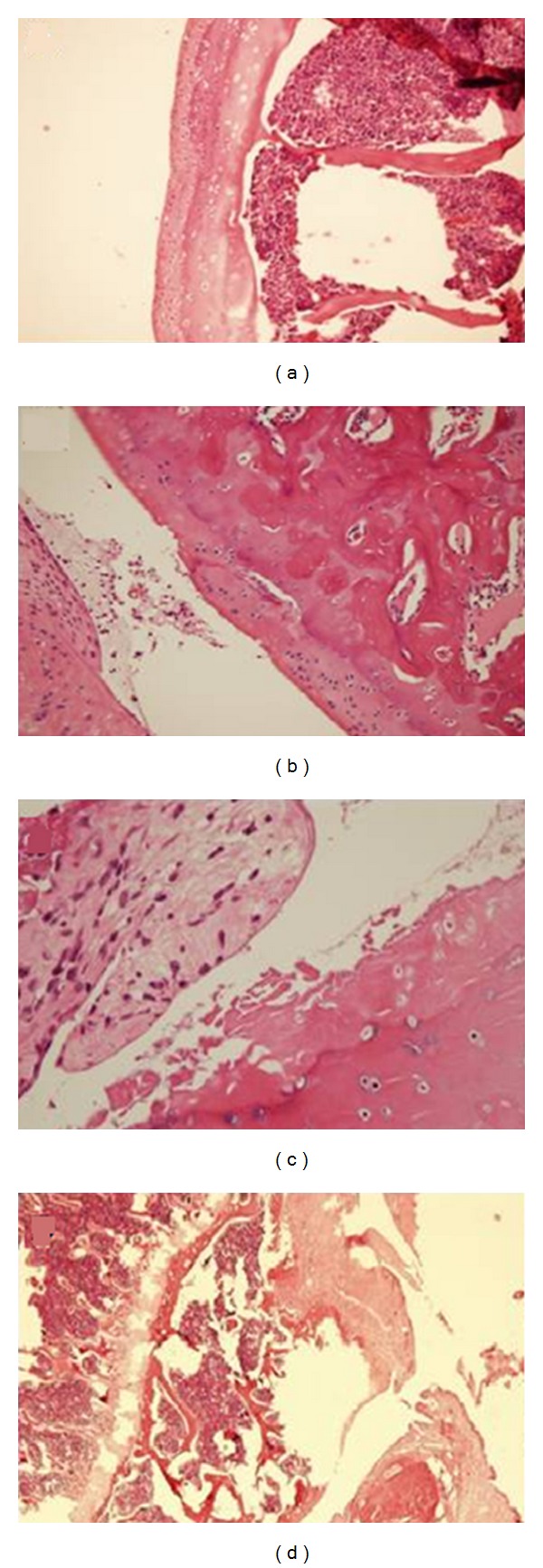
It is seen that complete layer of cartilage injury is histopathologically formed in the presence of H&E (×100). (a) Intact, (b) fibrillation, (c) fissure, and (d) osteophyte formation.

The formation of the cartilage complete layer injury was reported (Figure 3).

### 3.2. Statistical Evaluation

All of the evaluations were evaluated in 95% confidence interval and bilaterally. Alpha meaningfulness level was determined as ≤0.05; according to the specific activity results in synovial lavage fluid and cartilage tissue of the mediators (MMP-9, MMP-13, NO, TIMP-1, and TNF-*α*) emerging after complete layer articular cartilage injury.

No meaningful difference was determined in statistical terms between the TIMP-1 (*P* = 0.67) and MMP-9 (*P* = 0.28) levels in synovial lavage fluid and cartilage tissue. A meaningful difference was found in statistical terms between the MMP-13 (*P* < 0.0001), TNF-*α* (*P* < 0.0001), and NO (*P* < 0.0001) levels (Table 1). 

**Figure 4:**
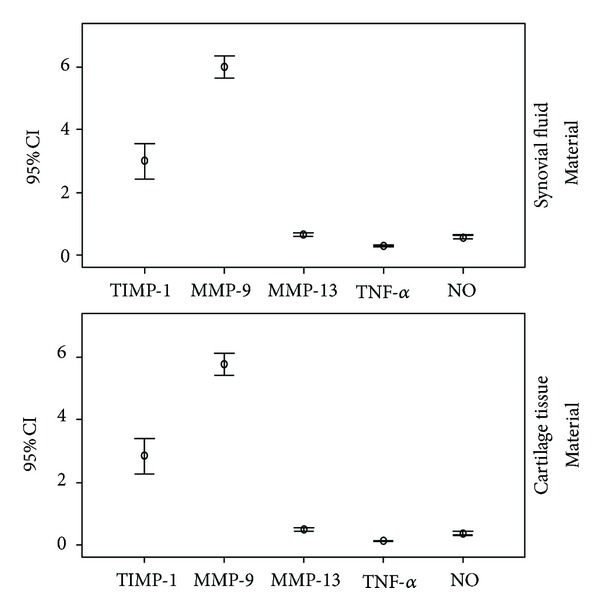
Distribution of the mediators in the synovial lavage fluid and cartilage tissue in all experimental groups (mean, 95% CI).

It was determined that the measurements in intragroup and intergroup TIMP-1 and MMP-9 mediators were variable, and close measurements were obtained in NO, TIMP-1, and TNF-*α* variables (Figure 4). 

In the partial correlation analysis performed, a negative linear meaningful relationship was found between TIMP-1 and MMP-13 (*P* < 0.0001, *r* = −0.38); a positive meaningful relationship was found between TIMP-1 and TNF-*α* (*P* < 0.0001, *r* = 0.33). A negative meaningful relationship was found between TIMP-1 and NO (*P* = 0.001, *r* = −0.26). And a positive linear meaningful relationship was found between MMP-9 and TNF-*α* (*P* < 0.0001, *r* = 0.34) and NO (*P* < 0.0001, *r* = 0.46) (Table 2). 

**Figure 5:**
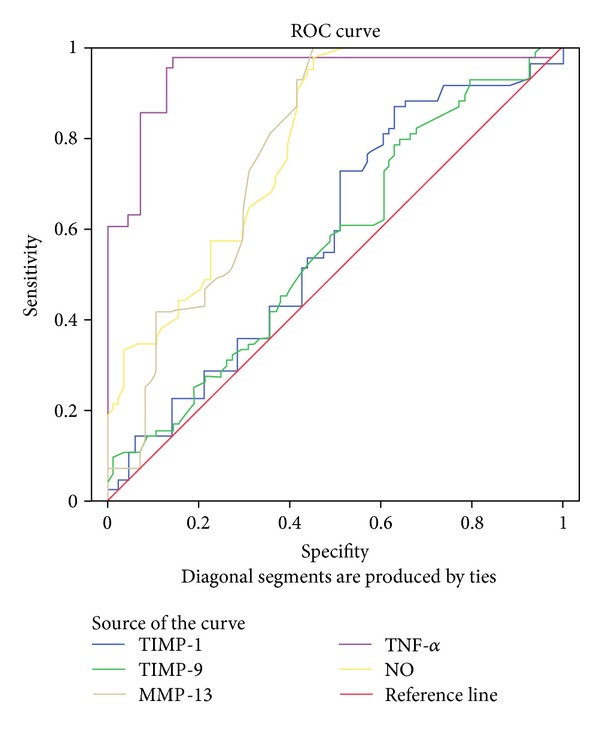
ROC analysis where the validities of the mediators in synovial lavage fluid and cartilage tissue is evaluated.

When the area under ROC curve of TIMP-1, MMP-9, MMP-13, NO, and TNF-*α* markers and *P* values were examined in the study groups, MMP-13 (*P* < 0.0001, 95% CI: 0.70–0.85), NO (*P* < 0.0001, 95% CI: 0.72–0.86), and TNF-*α* (*P* < 0.0001, 95% CI: 0.91–0.98), results were found to be statistically meaningful (Figure 5).

## 4. Discussion

Osteoartrit (OA) is a disease which emerges with the breakup of the balance between the construction and destruction of the cartilage in favour of destruction, results in the loss of the cartilage, and progresses slowly [18]. Recently, it has been shown that the MMPs whose levels in the joint at the time of injury increase play an important role in the destruction of the cartilage tissue [19]. One of the tissue inhibitors which enable the balancing of these enzymes which are destructive for the cartilage is identified as metalloproteinase [20]. Particularly, the metalloproteinase which increases in OA is produced by chondrocyte and synovial cells. Since the number of these cells is higher than that in the cartilage tissue, the main production source of MMP in the joint fluid is synovial tissue. It is shown that metalloproteinase and TIMP increased in the synovial fluid in OA and collagenase-TIMP-1 complex level generally stayed under the level which can be determined by immunoassay [21]. Lohmander et al. determined that metalloproteinase and TIMP levels stayed high for a long time in the knee joint after a traumatic injury [22].

In our study, it was seen that MMP-9 levels did not change even when different methodologies are used in synovial lavage fluid and cartilage tissue analyses in the samples which were examined after the formation of injury. Likewise, no change was determined in TIMP-1 levels (Table 1).

The role of proinflammatory was shown in the articular cartilage injury of TNF-*α* which are among the cytokines other than those aforementioned molecules [23, 24]. Serum TNF-*α* level is seen as a criterion of the synovial cell hyperactivity which becomes evident in the late stages of the cartilage injury [25]. It is known that TNF-*α* in the joint with OA mainly originates from the cells in the cartilage tissue, and these cells perform more cytokine expression when compared to synovial cells [26].

In our study, statistically meaningful differences were determined in terms of TNF-*α* and MMP-13 levels in the samples which were obtained from synovial lavage fluid and cartilage tissue (Table 1). In the subsequent partial correlation analysis, a negative meaningful relationship was found between TIMP-1 and MMP-13 (*P* < 0.0001, *r* = −0.38), and a positive meaningful relationship was found between TIMP-1 and TNF-*α* (*P* < 0.0001, *r* = 0.33). A positive linear meaningful relationship was found between MMP-9 and TNF-*α* (*P* < 0.0001, *r* = 0.34) (Table 2). It was seen in ROC analysis that these two mediators can also be used in different methodologies in synovial lavage fluid and cartilage tissue. The molecular marker level results which are obtained from different areas in the literature and set forth with different methodologies are also in conformity with the data of our study [25–27].

In an experimental study, it was it shown that NO synthesis are stimulated by several cytokines such as TNF-*α* [28]. Evidences regarding the fact that NO is a secondary fundamental mediator after TNF-*α* in cartilage cells were set forth in the same study [28]. In our study, on the other hand, NO level was determined to be meaningfully high in the analysis results of the samples taken from synovial lavage fluid in conformity with the TNF-*α* level in the experimental groups. A positive linear relationship was found between TNF-*α* and NO (*P* < 0.0001, *r* = 0.46). In the ROC analysis, the fact that these mediators which were taken from different places and examined can also be analysed over a different methodology cartilage tissue was confirmed.

The affect of MMP enzymes on cartilage is inhibited by TIMP [29, 30]. NO is overexpressed at synovial tissue of injured cartilage by the stimulus of inducible nitric oxide synthase and TNF-*α* [29–32]. IL-1 and TNF-*α* release is increased with the affect of degraded matrix molecules and so NO expression is also increased. It is reported that MMP-13 is discharged mostly via sinovial fluid [33]. We think that these data explain why TNF-*α*, MMP-13, and NO were found more in synovial fluids in our results.

We think the restriction of our study is that it cannot be crosschecked by a method other than ELISA method which is used in the analysis of the samples.

## 5. Conclusion

When, among the molecular markers which are analyzed in the synovial lavage fluid, MMP-9, MMP-13, NO, TIMP-1 and TNF-*α*, the area under the ROC curve and *P* values are evaluated, MMP-13 (*P* < 0.0001, 95% CI: 0.70–0.85), NO (*P* < 0.0001, 95% CI: 0.72–0.86), and TNF-*α* (*P* < 0.0001, 95% CI: 0.91–0.98) results are statistically significant. As a result, as it can be analyzed from the synovial lavage fluid of the molecular injury markers such as MMP-9, MMP-13, NO, TIMP-1, and TNF-*α* which emerge in the articular cartilage injury, we can say that it can also be analyzed in a reliable manner by the indirect ELISA method described by us in the samples which are obtained from the cartilage tissue. 

## Figures and Tables

**Table 1:** Evaluating MMP-9, MMP-13, NO, TIMP-1 and TNF-alpha levels in synovial lavage fluid and cartilage tissue types.

Material		*N*	Mean	Standard deviation	*P*
TIMP-1	Synovial lavage fluid	84	3.01	2.61	0.67
	Cartilage Tissue	84	2.83	2.59	
MMP-9	Synovial lavage fluid	90	5.92	1.63	0.28
	Cartilage Tissue	90	5.66	1.67	
MMP-13	Synovial lavage fluid	90	0.68	0.23	<0.001
	Cartilage Tissue	90	0.47	0.24	
TNF-*α*	Synovial lavage fluid	84	0.32	0.09	<0.001
	Cartilage Tissue	84	0.14	0.07	
NO	Synovial lavage fluid	90	0.57	0.19	<0.001
	Cartilage Tissue	90	0.36	0.19	

**Table 2:** Partial Correlation analysis.

Control variables	TIMP-1	MMP-9	MMP-13	TNF-*α*	NO
Material					
TIMP-1					
Correlation	1.00	−0.14	−0.38	0.33	−0.26
Significance (2-tailed)	—	0.08	0.000	0.000	0.001
MMP-9					
Correlation	−0.14	1,0	−0.04	0.34	0.46
Significance (2-tailed)	0.08	—	0.61	0.000	0.000
MMP-13					
Correlation	−0.38	−0.04	1.000	0.13	0.01
Significance (2-tailed)	0.000	0.61	—	0.09	0.88
TNF-*α*					
Correlation	0.33	0.34	0.13	1.000	−0.04
Significance (2-tailed)	0.000	0.000	0.09	—	0.59
NO					
Correlation	−0.26	0.46	0.01	−0.04	1.000
Significance (2-tailed)	0.001	0.000	0.88	0.59	—
